# Virion-Associated Cholesterol Regulates the Infection of Human Parainfluenza Virus Type 3

**DOI:** 10.3390/v11050438

**Published:** 2019-05-15

**Authors:** Qiaopeng Tang, Pengfei Liu, Mingzhou Chen, Yali Qin

**Affiliations:** State Key Laboratory of Virology and Modern Virology Research Center, College of Life Sciences, Wuhan University, Wuhan 430072, China; tangqiaopeng@163.com (Q.T.); 2016202040031@whu.edu.cn (P.L.)

**Keywords:** HPIV3, fusion protein, viral assembly, lipid rafts, cholesterol, internalization

## Abstract

The matrix (M) proteins of paramyxoviruses bind to the nucleocapsids and cytoplasmic tails of glycoproteins, thus mediating the assembly and budding of virions. We first determined the budding characterization of the HPIV3 Fusion (F) protein to investigate the assembly mechanism of human parainfluenza virus type 3 (HPIV3). Our results show that expression of the HPIV3 F protein alone is sufficient to initiate the release of virus-like particles (VLPs), and the F protein can regulate the VLP-forming ability of the M protein. Furthermore, HPIV3_F-Flag_, which is a recombinant HPIV3 with a Flag tag at the C-terminus of the F protein, was constructed and recovered. We found that the M, F, and hemagglutinin-neuraminidase (HN) proteins and the viral genome can accumulate in lipid rafts in HPIV3_F-Flag-_infected cells, and the F protein mainly exists in the form of F_1_ in VLPs, lipid rafts, and purified virions. Furthermore, the function of cholesterol in the viral envelope and cell membrane was assessed via the elimination of cholesterol by methyl-β-cyclodextrin (MβCD). Our results suggest that the infectivity of HPIV3 was markedly reduced, due to defective internalization ability in the absence of cholesterol. These results reveal that HPIV3 might assemble in the lipid rafts to acquire cholesterol for the envelope of HPIV3, which suggests the that disruption of the cholesterol composition of HPIV3 virions might be a useful method for the design of anti-HPIV3 therapy.

## 1. Introduction

Human parainfluenza virus type 3 (HPIV3) is an enveloped, non-segmented, negative, single-strand RNA virus, and it acts as one of the primary pathogens that causes respiratory tract diseases, including bronchiolitis, pneumonia, and croup in infants and young children. The genome of HPIV3 encodes six structural proteins: the nucleoprotein (N), polymerase cofactor phosphoprotein (P), matrix (M) protein, fusion (F) protein, hemagglutinin-neuraminidase (HN) protein, and polymerase large protein (L). The binding of the HN protein to cellular sialic acid-containing receptors induces a conformational change of the F protein early in the infection of HPIV3, which mediates the fusion of the viral envelope with the cell membrane [1]. Finally, the viral genome and N, P, and L proteins are released into the cytoplasm for transcription and replication. Despite the fact that many details of the paramyxovirus life cycle are well known, the mechanism of virion assembly and budding are less well characterized.

Lipid rafts seem to be their preferred sites for budding during their life cycles for many viruses, as lipid rafts contain abundant cholesterol, sphingolipid, and proteins, which allow them to act as platforms that function in cell membrane signaling [2] and trafficking [3]. Several functions of lipid rafts in multiple steps of the life cycle of many different viruses have been reported. First, lipid rafts can mediate the process of entry, as the lipid rafts can increase the local concentration of entry receptors to increase the human immunodeficiency virus type 1 (HIV-1)’s binding efficiency and fusion events [4]. Many cellular receptors of viruses are associated with lipid rafts, and the association mediates raft-dependent endocytosis and helps in virus entry; the infection of simian virus 40 [5] and echovirus type 1 [6] comply with this model. Second, lipid rafts can aggregate viral components to promote virus assembly; Hemagglutinin (HA) and Neuraminidasee (NA) of influenza virus [7,8], the F protein of measles virus [9] and respiratory syncytial virus (RSV) [10], the RSV SH protein [11], the F and HN proteins of Sendai virus [12], the F protein of Newcastle Disease Virus (NDV) [13], the Env glycoprotein of HIV-1 [14], and the genome of Tiger Frog Virus [15] have been reported to be associated with lipid rafts. Third, lipid rafts can also be critical in virus budding, as the cultivation of human T lymphocytes in cholesterol-poor medium that contains the HMG-Coenzyme A reductase inhibitor, lovastatin, can reduce the release of HIV-1 [16]; viperin can inhibit influenza virus release by the interaction with farnesyl diphosphate synthase, which affects the formation of lipid rafts [17]; and, Methyl-β-cyclodextrin (MβCD) can reduce the attachment of NDV proteins with lipid rafts and it results in an enhanced release of the virus with reduced infectivity [18]. Due to the critical role of lipid rafts as platforms for virus assembly and budding, the envelopes of many viruses contain abundant cholesterol and sphingomyelin. Increasing evidence shows that virion-associated cholesterol and sphingolipid are critical in virus entry, especially in the fusion stage between the virus envelope and the cell membrane. Thus, MβCD treatment of Human herpesvirus 6, Borna disease virus, Dengue virus, and Hepatitis C Virus leads to the dramatic reduction of viral infectivity [19,20,21,22]. Most likely, the reduction of cholesterol in the viral envelope causes a change in the relative position of the F and HN proteins, which thereby affects the interaction between the F and HN proteins, and eventually the F protein cannot no longer be activated by the HN protein to produce conformational changes that are essential in the fusion of the viral envelope with the cell membrane. However, where and how HPIV3 is assembled and whether the lipid rafts participate in the life cycle of HPIV3 remains unknown.

In this study, we found that the F protein of HPIV3 can form virus-like particles (VLPs) when expressed alone, and the F protein can regulate the ability of the M protein to form VLPs, which suggests that the F protein also plays an important role in the assembly and budding of HPIV3. Furthermore, our results show that most of the F protein and the HN protein and a small part of the M protein and virus genome partitioned into cellular lipid rafts in HPIV3-infected cells. F_1_ were found to be the main form in VLPs, lipid rafts, and virions, which indicated that HPIV3 might utilize lipid rafts for assembly and budding. Finally, we found that HPIV3 envelope-associated cholesterol affects the fusion between the virus and the cell membrane. These results suggest that HPIV3 may acquire envelope associated-cholesterol from lipid rafts for virus infection, which provides us with a new strategy for anti-HPIV3 therapy. However, the mechanism of how viral envelope-associated cholesterol affects membrane fusion requires further research.

## 2. Materials and Methods

### 2.1. Cells and Virus

Hela, MK2, and 293T cells were cultured in Dulbecco’s modified Eagle’s medium (DMEM; Gbico, Carlsbad, CA, USA) that was supplemented with 10% fetal bovine serum (FBS). HPIV3 (NIH47885) and HPIV3_F-Flag_ were amplified in MK2 cells by inoculation at a multiplicity of infection (MOI) of 0.1.

### 2.2. Plasmid Constructs

The plasmids pCAGGS-Flag-F-Flag and pCAGGS-HA-HN were generated by PCR-based cloning techniques and then cloned into pCAGGS. pOCUS-HPIV3 was used as a template for all of the genetic manipulations. Plasmids carrying the HPIV3 genome with a Flag tag fused to the C terminus of the *F* gene were constructed, as described previously [23]. All of the plasmids were verified by DNA sequencing.

### 2.3. VLP Budding Assay

293T cells in 6 cm plates were grown to 50%–60% confluence and transfected with the plasmids indicated below. Empty pCAGGS plasmids were used to equalize the DNA amount for transfections. At 36 h, the post-transfection cells and the culture medium were collected and centrifuged, as described previously [24].

### 2.4. Protease Protection Assay

VLPs from the medium of cells that were transfected with Flag-tagged wild-type F were prepared as described above. Four aliquots were treated as described previously [24]. Subsequently, samples were mixed with SDS-PAGE loading buffer and boiled for Western analysis.

### 2.5. Immunofluorescence and Confocal Microscopy

Hela cells in 12-well plates were cultured on glass coverslips. The plasmids’ DNA indicated below were transfected by using Lipofectamine 2000 (Invitrogen, Carlsbad, CA, USA) when the cell confluency grew to 50%–60%. At 24 h post-transfection, the cells were washed three times with cold Phosphate buffer saline (PBS), fixed with 0.4% paraformaldehyde, and permeabilized with 0.2% Triton X-100 for 20 min. at room temperature. The permeabilized cells were blocked for 1 h in PBS, supplemented with 3% bovine serum albumin (BSA) at room temperature, followed by the primary mouse monoclonal anti-Flag antibody (Sigma; 1:1000) in blocking buffer for 2 h at 4 °C, followed by goat anti-mouse IgG fluorescein secondary antibody (Thermo; 1:200) for 45 min. at room temperature. After being washed three times with cold 1% BSA, the coverslips were turned over and mounted onto one drop of histology mounting medium (Fluoroshield with 4′,6-diamidino-2-phenylindole (DAPI); Sigma-Aldrich, St. Louis, MO, USA) on glass slides. Confocal images were collected to detect the location of F using an Olympus confocal FV 1000 microscope. 

### 2.6. Transfection and Recovery of Recombinant HPIV3_F-Flag_

293-T7 cells in six-well plates, grown to 40% confluence, were transfected with PGEM4-N (400 ng), PGEM4-P (400 ng), PGEM4-L (200 ng), and Pocus-HPIV3_F-Flag_ (4 μg) via calcium phosphate transfection at 37 °C. Recombinant HPIV3 was recovered, as described previously [25].

### 2.7. Raft Flotation Assay

293T cells that were cultured in 175 mm were transfected with HPIV3 proteins or infected with HPIV3_F-Flag_. The cells were harvested by scraping and pelleted by low-speed centrifugation in an Eppendorf centrifuge (4000 rpm for 3 min) at 4 °C and then lysed in 2 mL of cold TNE buffer (50 mM Tris (pH 7.4), 150 mM NaCl, 5 mM Ethylenediaminetetraacetic acid disodium salt (EDTA) containing 1% Triton-X 100 on ice for 30 min. The cell lysates were centrifuged at 4000 rpm for 10 min. at 4 °C. Each clarified supernatant (2 mL) was mixed with 2 mL of 80% sucrose in TNE buffer containing 1% Triton-X 100 to a final sucrose concentration of 40%. Subsequently, 3.66 mL of the mixture was placed at the bottom of the 12-mL ultracentrifuge tube and overlaid with 4.58 mL of 35% sucrose and 2.75 mL of 5% sucrose in TNE buffer containing 1% Triton-X 100. 11 1-mL fractions were collected and subjected to trichloroacetic acid precipitation after centrifugation at 35,000 rpm for 16 h at 4 °C in a P40ST rotor (Hitachi, Tokyo, Japan). The concentrated samples were mixed with SDS-PAGE loading buffer and then boiled at 100 °C for 10 min. The proteins in each layer were detected by sodium dodecyl sulfate polyacrylamide gel electrophoresis with anti-HA, anti-Flag, anti-Myc, anti-HN, anti-CD71, and anti-Caveolin-1 as the primary antibodies.

### 2.8. Cholesterol Extraction and Measurement

293T cells in 12-well plates, grown to 70%–90%, were resuspended with 1 mL of cold PBS and then pelleted by centrifugation at 13,000 rpm for 1 min. at 4 °C. Afterwards, 600 μL of mixed solution of trichloromethane and methanol in a volume ratio of 2 to 1 were added to the pelleted cells. After being incubated in a shaker at 250 rpm for 2 h at 37 °C, 400 µL of deionized water was added, the solution was mixed on a Vortex Oscillator for 10 s, and then left standing still for 10 min. The mixed solution was centrifuged at 13,000 rpm for 10 min, and then the organic phase under the solution was transferred into a new tube. The organic phase was evaporated at 55 °C for 3 h. Subsequently, 200 µL of the reaction buffer was added to the bottom of the tube to resuspend the cellular cholesterol. The cholesterol in 293T cells was measured by using the Amplex^TM^ Red Cholesterol Assay Kit, according to the manufacturer’s protocol. For the measurement of virus cholesterol, HPIV3 virions, which were purified through a cushion of 20% sucrose was used, as the pelleted cells were subjected to the same steps to extract cholesterol.

### 2.9. Virus Infection and Plaque Assay

MK2 cells that were cultured in 24-well plates with 60%–70% confluency were infected with MβCD-pretreated or untreated HPIV3 at an MOI of 0.05 PFU/cell for 30 min. at 37 °C with 5% CO_2_; then, the virus was removed and replaced with fresh DMEM containing 10% FBS. For the plaque assay, following the infection procedure, a virus-containing culture was serially diluted 10-fold up to 10^5^. MK2 cells in 24-well plates with 60%–70% confluence were infected with 200 µL of the dilutions. After infection for 2 h at 37 °C, the virus-containing medium was replaced with methylcellulose, the cell plates were incubated for another 5–7 days to detect any visible viral plaques, and finally the plaques were counted using 0.5% crystal violet staining.

### 2.10. qRT-PCR Assay

Cell and virus RNA was extracted by TRIzol and reverse transcripted with primers Oligo(dt) and HPIV3-N-R(5′-3′AATTCCATACCTGATTGTATT). Subsequently, q-PCR was done with Quant one step qRT-PCR Kit SYBR Green kits from TIANGEN with primers GAPDH-F(5′-3′CTCTGCTCCTCCTGTTCGAC), GAPDH-R(5′-3′CGCCCGCGTCCGGCCTACACA), HPIV3-N-R(5′-3′AATTCCATACCTGATTGTATT), and HPIV3-N-F(5′-3′ATCAGATTGGGTCAATCAT), according to the manufacturer’s protocol.

### 2.11. Methyl-β-Cyclodextrin Treatment of Cells and Virions

293T cells in 12-well plates were grown to 70%–90% confluency and washed twice with medium containing no FBS, and then 1 mL of media with final concentrations of MβCD of 0 mM, 2 Mm, 5 mM, and 10 mM were added in the plates at 37 °C for 30 min. After treatment with MβCD, the cells were washed twice with fresh medium containing 10% FBS. For treatment HPIV3 virions, 1 mL of cell-free HPIV3 virus were added to MβCD at different final concentrations at 37 °C for 30 min, layered onto a cushion of 20% (wt./vol) sucrose in PBS, and subsequently ultracentrifuged on a P55ST2 rotor (Hitachi) at 35,000 rpm for 1 h at 4 °C to remove MβCD. The purified virus was resuspended in 50 µL PBS for cell infection.

### 2.12. Virus Binding and Internalization Assays

293T cells were cultured at 1 × 10^5^ cells/well in six-well plates for 24 h before infection. To measure binding ability, the cells were incubated for 1 h at 4 °C before exposure to MβCD-pretreated HPIV3 virions. After 1 h of infection, the cells were washed five times with ice-cold PBS, and then cells were collected by centrifugation at 13,000 rpm for 1 min, followed by lysis with 1 mL of TRIzol reagent (Invitrogen) to extract the total RNA for RT-PCR detection. To measure internalization, following the virus binding procedure, 293T cells were warmed to 37 °C and maintained for 2 h, after which the cells were treated with 0.25% trypsin for 5 min. at 37 °C.

## 3. Results

### 3.1. The HPIV3 F Protein Alone Is Sufficient to Release VLPs

**Figure 1:**
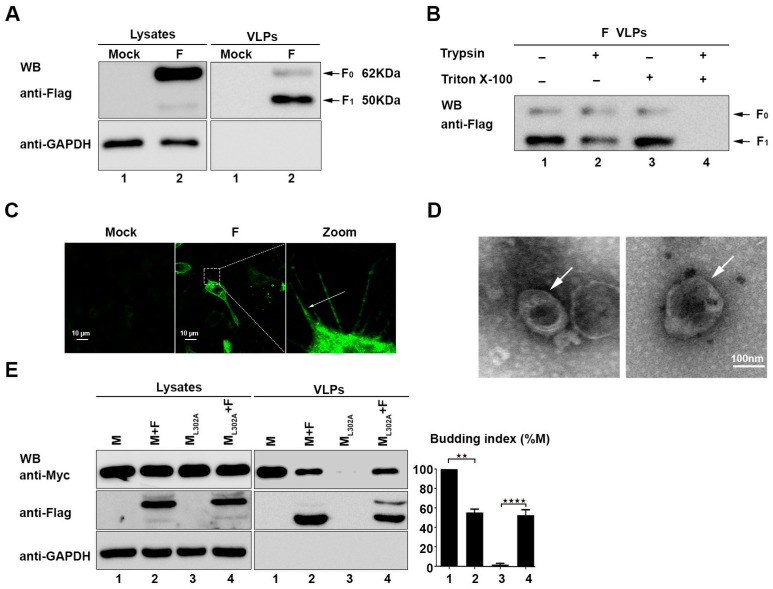
The human parainfluenza virus type 3 (HPIV3) Fusion (F) protein alone can secrete into the supernatant from transfected cells and form virus-like particles (VLPs). (**A**) 293T cells were transfected with the indicated plasmids for 36 h. The cell lysates and VLPs were prepared as described in Section 2, and then, the samples were subjected to western blot analysis (WB) by using anti-Flag and anti-GAPDH antibodies (Abs). Arrows indicate the bands of the detected proteins. (**B**) A protease protection assay of the HPIV3 F protein VLPs was performed, as described in Section 2 and then analyzed via WB with an anti-Flag antibody. (**C**) Cellular localization of the HPIV3 F protein. Hela cells were transfected with the plasmid encoding wild-type F, fixed 24 h after transfection, stained with an anti-Flag Ab, and visualized via confocal microscopy as describe in Section 2. Arrows indicate the filamentous structure formed by the F protein. (**D**) Representative TEM graphs of the F protein VLPs. VLP samples were prepared as previously described and then visualized by transmission electron microscopy. (**E**) The F protein was transfected with M/ML302A in 293T cells, and 36 h post-transfection, the cell lysates and VLPs were prepared as described in Section 2. Then, the samples were subjected to western blot analysis by using anti-Flag, anti-GAPDH, and anti-Myc Abs. Arrows indicate the bands of the detected proteins. Error bars, mean ± SD of three experiments. Student’s t test; * *p* < 0.05; ** *p* < 0.01; *** *p* < 0.001; **** *p* < 0.0001; NS, non-significant.

Previous studies showed that the F protein of nipah [26] virus and hendra [27] virus can release VLPs when expressed alone. Thus, we wanted to determine whether the F protein of HIPV3 also bears a similar function. To explore detailed assembly and budding processes of HPIV3, the plasmids of the HPIV3 F protein with a flag tag at the C terminal were constructed and transfected into 293T cells. Subsequently, 36 h after transfection, cell supernatants were collected and subjected to ultracentrifugation through a 20% (wt./vol) sucrose cushion to pellet VLPs for western blotting (WB). The result of WB with the anti-Flag antibody showed that two prominent bands with apparent molecular masses of 62 kDa (F_0_) and 50 kDa (F_1_) were detected in the lysates and VLPs (Figure 1A). Interestingly, F_0_ was the main product in the cell lysates, but F_1_ was the main cleavage product in the VLPs. To further examine whether VLPs are present in the membrane-bound form, a protease protection assay of VLPs was performed. The purified VLPs of the HPIV3 F protein were left untreated or treated with Triton X-100, trypsin, or Triton X-100 plus trypsin. In the VLPs that were treated with trypsin alone, no significant digestion of the F protein was observed when compared with the untreated samples. The F protein remained unchanged when it was treated with Triton X-100 alone. However, when treated with trypsin plus Triton X-100, F_0_ and F_1_ in the VLPs were completely degraded (Figure 1B), which suggested that the F protein was released into the culture medium in a membrane-protected form. In addition, our previous studies showed that the expression of the HPIV3 wild-type M protein, but not M_L302A_ (a mutant of the M protein that is unable to form VLPs), can form a filamentous structure at the edge of the cell membrane [24], which indicates that the formation of the filamentous structure at the edge of the cell membrane is auxiliary evidence for evaluating whether a transfected protein can form VLPs. Therefore, immunofluorescence experiments were performed to locate the F protein in the transfected cells, and we found that the filamentous structures formed by the expression of the F protein are similar to that formed by the M protein (Figure 1C). Furthermore, the morphology of the F protein VLPs, determined by electron microscopy (Figure 1D), was also similar to that of the M protein and virions of HPIV3, as previously described [24]. The above results show that the F protein expression alone is sufficient to release VLPs.

### 3.2. The F Protein Regulates VLP Formation and Release of the M Protein

Knowing that the respective expression of the F and M proteins of HPIV3 alone can release VLPs, we co-expressed the M protein or the M_L302A_ protein with the F protein, respectively, to investigate the relationship between the F and M proteins. We found that the ability of the wild-type M protein to form VLPs was inhibited in the presence of the F protein, while the VLP formation ability of M_L302A_ was partially rescued by co-expression with the F protein (Figure 1E). These results suggest that the F protein plays a critical role in the virion assembly of HPIV3 by association with the M protein and the regulation of the release of the M protein.

### 3.3. Recovery of Recombinant HPIV3_F-Flag_

**Figure 2:**
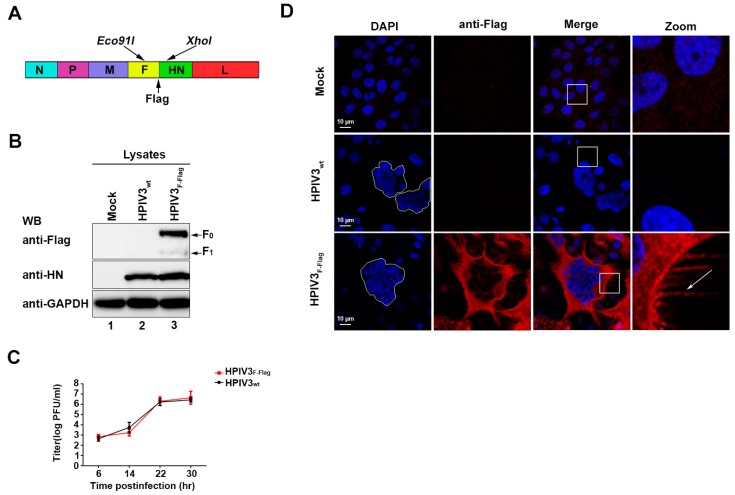
Recovery of recombinant HPIV3_F-Flag_. (**A**) Recombinant virus HPIV3_F-Flag_ construction mode. (**B**) Hela cells were mock-infected or infected with wild-type HPIV3 or HPIV3_F-Flag_. At 24 h post-infection, cell lysates were subjected to WB as described in Section 2 with anti-Flag, anti- hemagglutinin-neuraminidase (HN), and anti-GAPDH Abs. (**C**) The growth properties of HPIV3 and HPIV3_F-Flag_. MK2 cells were infected with HPIV3 or HPIV3_F-Flag_ at a multiplicity of infection (MOI) of 1; titers of supernatants that were harvested at 6, 14, 22, and 30 h after virus infection were examined. (**D**) Cellular localization of the HPIV3_F-Flag_ F protein. Hela cells were mock-infected or infected with wild-type HPIV3 or HPIV3_F-Flag_. At 24 h post-infection, the cells were stained with anti-Flag and visualized via confocal microscopy, as described in Section 2. The arrows indicate the filamentous structure formed by HPIV3_F-Flag_.

Due to the F protein of HPIV3 playing a key role in the formation of HPIV3 particles, studying the localization of the F protein in HPIV3-infected cells is important in exploring the mechanism of HPIV3 assembly and budding. Therefore, a recombinant HPIV3 with a Flag tag at the C-terminal of the F protein was constructed and rescued by using reverse genetics. Figure 2A shows the construction mode. In order to verify the recombinant HPIV3 virus, western blot was performed to detect Flag signals in the lysates of cells that were mock-infected, HPIV3-infected, and HPIV3_F-Flag_-infected. Our results show that Flag-tagged F_0_ and F_1_ can be clearly detected in HPIV3_F-Flag_-infected cells, but not mock-infected or HPIV3-infected cells. Although, with nearly same expression level of the HN protein in HPIV3-infected and HPIV3_F-Flag_-infected cells, these results indicate that a Flag tag was successfully added to the C-terminal of the F protein in HPIV3 (Figure 2B). Simultaneously, we also found that the addition of a Flag tag to the C-terminal of the F protein did not affect the virus growth ability (Figure 2C). When considering the possibility that the F protein may aggregate at the virions’ assembly and budding site in virus-infected cells, immunofluorescence was performed to detect the location of the F protein in the HPIV3_F-Flag_-infected cells. Similar to the result of WB, the immunofluorescence results show that a Flag tag signal can only be detected in cells that were infected with HPIV3_F-Flag_. Visible syncytia were also observed in the HPIV3- and HPIV3_F-Flag_-infected cells, but not in the mock-infected cells (Figure 2D). Although we did not find any aggregation of the F protein in HPIV3_F-Flag_-infected cells, interestingly a filamentous structure that was formed by the F protein was observed at the edge of the plasma membrane in HPIV3_F-Flag_-infected cells. Nevertheless, the functions of those filamentous structures in the life cycle of HPIV3 remain to be further studied.

### 3.4. The F Protein, HN Protein, and Virus Genome Localized in Lipid Rafts in HPIV3_F-Flag_-Infected Cells

**Figure 3:**
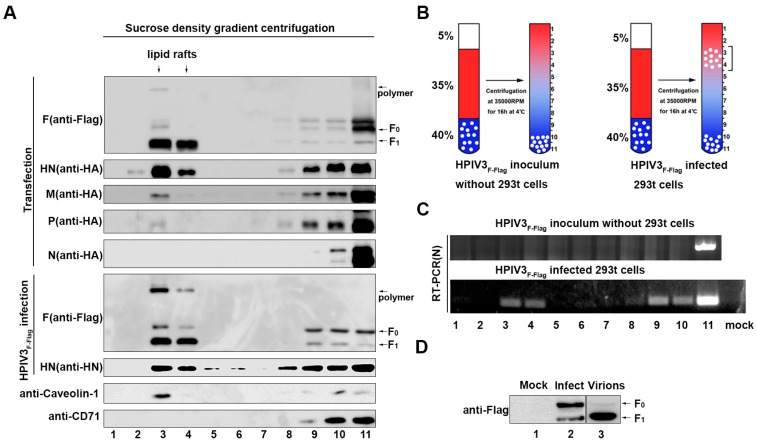
The F, HN, and matrix (M) proteins and virus genome accumulate in lipid rafts. (**A**) 293T cells were transfected with F, HN, M, N, and P proteins or infected with HPIV3_F-Flag_. Afterwards, 36 h post-transfection or 24 h post-infection, the cells were extracted with 1% Triton X-100 at 4 °C. The lysates were loaded at the bottom of a flotation sucrose density gradient and subjected to equilibrium centrifugation. The gradient was fractionated from the top, and samples were analyzed by WB with anti-Flag, anti-HA, anti-Myc, anti-HN, anti-Caveolin-1, and anti-CD71 Abs. (**B**) The mode that purified HPIV3_F-Flag_ virions or HPIV3_F-Flag_-infected cells were loaded at the bottom of a flotation sucrose density gradient. (**C**) Extracted RNA from each fraction was amplified targeting the HPIV3 *N* gene by RT-PCR and then examined. (**D**) The F protein in mock-infected, HPIV3_F-Flag_-infected cells, and purified virions. MK2 cells were mock-infected or HPIV3_F-Flag_-infected at a MOI of 0.1. At 36 h post-infection, cells supernatants were subjected to ultracentrifugation through a 20% sucrose cushion to pellet the virions, and cells were lysed, as described in Section 2.

It has been established that lipid rafts can act as an assembly and budding site for many viruses [14,15,28]. For these viruses, glycoproteins and viral genomes can usually be enriched in lipid rafts. Therefore, in order to verify whether the lipid rafts have such a role in the life cycle of HPIV3, we infected 293T cells with HPIV3_F-Flag_ or separately expressed the F protein, HN protein, M protein, N protein, and P protein in 293T cells, and the cells were lysed with a lysis buffer containing 1% Triton X-100 at 4 °C for 30 min. The lipid rafts were separated by sucrose density gradient centrifugation. The virus genome RNA in each separation layer, which was acquired from cryogenic ultracentrifugation, was examined by RT-PCR. Endogenous caveolin-1 protein was used as a marker of lipid rafts, and CD71 was used as a marker for non-lipid rafts. The results show that most of the F and HN proteins were enriched in lipid rafts; only a small fraction of the M and P proteins were localized in lipid rafts, while none of the N protein was observed in the layer of lipid rafts in the plasmid-transfected cells (Figure 3A). Correspondingly, large amounts of the F and HN proteins were also detected in lipid rafts in HPIV3_F-Flag_-infected cells (Figure 3A), whereas the M protein, N protein, and P protein were not examined due to the lack of specific antibodies, which suggests that HPIV3 is likely to use lipid rafts to enrich viral proteins for virus assembly and budding. To further confirm whether the genome RNA of HPIV3_F-Flag_ is also located in lipid rafts, the HPIV3-infected cells were examined by RT-PCR with specific primers targeting the *N* gene (Figure 3B). The result show that part of the viral genome in HPIV3_F-Flag_-infected cells moved from the bottom to the higher lipid raft layers (Figure 3C), which suggests that HPIV3 nucleocapsids localize in lipid rafts in virus-infected cells. Furthermore, we found that whether in VLP or in lipid rafts, the F proteins were mainly in the form of F_1_, while, in cell lysates, the F protein mainly exists in the form of F_0_. Meanwhile, the existing form of the F protein in HPIV3_F-Flag_ and purified virions was mainly in the form of F_1_ (Figure 3D). These results suggest that HPIV3 may assemble and bud from lipid rafts.

### 3.5. Disruption of Lipid Rafts from Cellular Membranes Does Not Prevent HPIV3 Infection and Budding

**Figure 4:**
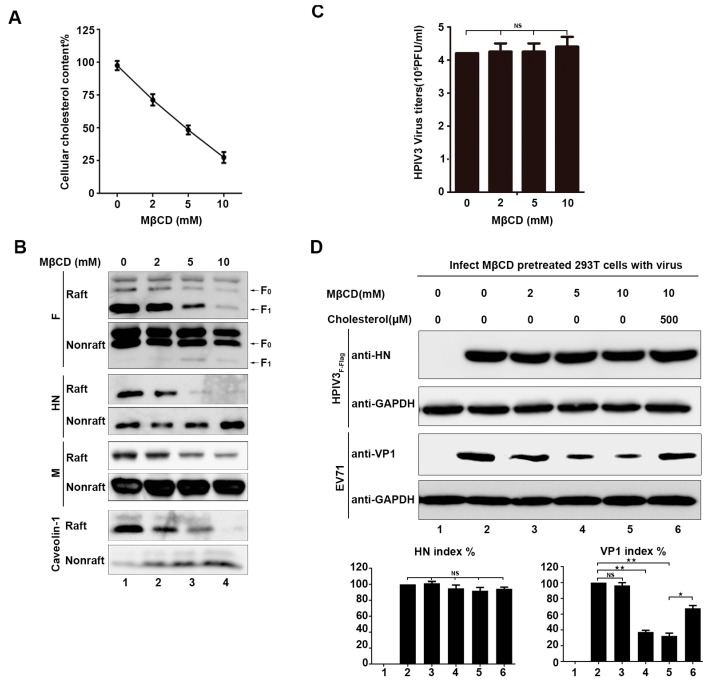
Destruction of lipid rafts of cellular membranes did not prevent HPIV3 infection and budding. (**A**) 293T cells were incubated with various concentrations of methyl-β-cyclodextrin (MβCD) at 37 °C. Subsequently, 30 min. after treatment with MβCD, cellular cholesterol was measured by an Amplex^TM^ Red Cholesterol Assay Kit according to the manufacturer’s protocol. (**B**) 293T cells were transfected with the indicated plasmids. Then, 36 h post-transfection, the cells were incubated with various concentrations of MβCD at 37 °C. Next, 30 min. after treatment with MβCD, the cells were extracted with 1% Triton X-100 at 4 °C. The lysate was loaded at the bottom of a flotation sucrose density gradient and subjected to equilibrium centrifugation. Fractions of lipid rafts and non-lipid rafts were analyzed by WB with anti-Flag, anti-HA, anti-Myc, and anti-Caveolin-1 Abs. (**C**) 293T cells were infected with HPIV3 at a MOI of 0.1. Afterwards, 12 h post-infection, the cells were treated with the indicated MβCD for 30 min, and 12 h after treatment with MβCD, the supernatant of the cells was collected, and the virus titers were measured. (**D**) 293T cells were incubated with MβCD and cholesterol as indicated. Next, the cells were infected with HPIV3_F-Flag_ or EV71. Then, 12 h post-infection, cell lysates were subjected to WB as described in Section 2 with anti-HN, anti-VP1, and anti-GAPDH Abs. Error bars, mean ± SD of three experiments. Student’s *t* test; * *p* < 0.05; ** *p* < 0.01; *** *p* < 0.001; **** *p* < 0.0001; NS, non-significant.

Knowing that the virus genome RNA, F protein, and HN protein can move into the lipid raft layer in virus-infected cells, the potential function of lipid rafts in the life cycle of HPIV3 was detected by treating 293T cells with various concentrations of MβCD, which is a drug that can destroy lipid rafts of cell plasma membrane by effectively depriving them of cholesterol [29]. First, we examined the total cholesterol content of cells that were treated with various concentrations of MβCD using an Amplex^TM^ Red Cholesterol Assay kit. The results show that, when the concentration of MβCD reaches 5 mM, the total cellular cholesterol content has been reduced by half, and, when the concentration of MβCD reaches 10 mM, the total cell cholesterol content has decreased by about 75% (Figure 4A). Second, in order to prove that MβCD can indeed destroy lipid rafts, 293T cells that were transfected with the F protein, HN protein, and M protein were exposed to increasing concentrations of MβCD, and the distribution of viral proteins and endogenous Caveolin-1 in the lipid raft layers were examined by WB. The results show that, as the MβCD concentration increased, the viral proteins and endogenous Caveolin-1 gradually decreased in lipid rafts, which suggests that MβCD can effectively destroy lipid rafts (Figure 4B). Subsequently, we analyzed the generation of HPIV3_F-Flag_ virions that were treated with different concentrations of MβCD for 30 min. at 37 °C. We added serum-free medium to the cell culture dish after adding cells treated with MβCD in order to avoid the effect of cholesterol in the culture medium on the function of lipid rafts, and 6 h post-infection, the supernatants were collected to detect viral titers. The results show that there is no effect on viral budding when cells are treated with MβCD, suggesting that viruses released from MβCD-treated cells still have sufficient cholesterol, which demonstrates that HPIV3 does not directly utilize the lipid rafts of cell membranes for assembly and budding (Figure 4C). Afterwards, EV71 was taken as a positive control, for which it has been reported that the entry of a virus is dependent on the cell membrane lipid rafts to detect whether damage to cell membrane lipid rafts affects HPIV3 infection [30]. We treated cells with MβCD before infection, and. at 24 h post-infection, the cells were lysed, as described in Section 2 and subjected to western blot with anti-HN and anti-GAPDH antibodies (Abs). Our results show that the destruction of cell membrane lipid rafts with MβCD affects the infection of EV71, but not HPIV3, which means that the infection of HPIV3 is not dependent on cell membrane lipid rafts (Figure 4D).

### 3.6. Depletion of Viral Envelope Cholesterol Markedly Reduces Infectivity of HPIV3

**Figure 5:**
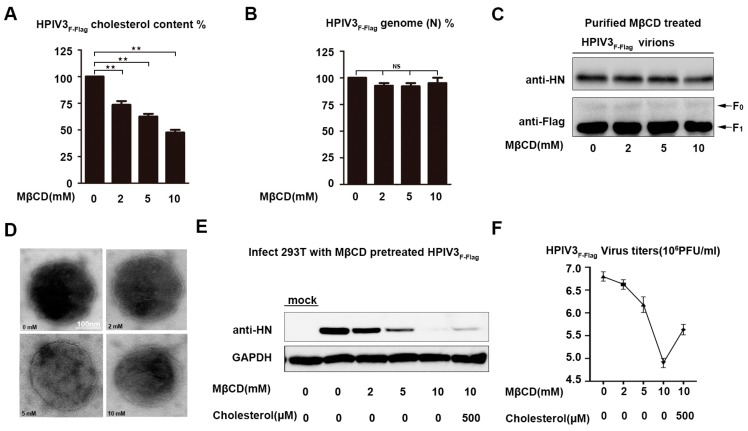
Infectivity of cholesterol-depleted HPIV3 was markedly reduced. (**A**) HPIV3_F-Flag_ virus was pretreated with various concentrations of MβCD or left untreated, and then, the virions were repurified using a sucrose cushion in order to analyze the cholesterol content using an Amplex^TM^ Red Cholesterol Assay Kit according to the manufacturer’s protocol. (**B**) The relative number of the genomes of the sucrose-cushion-repurified virus that was treated with MβCD and cholesterol were detected by Q-PCR. (**C**) The number of the HN and F proteins on the repurified MβCD-treated HPIV3 virions were detected by WB. (**D**) Electron microscope image of MβCD-treated virus. The sucrose-cushion-repurified HPIV3 virus that was treated with MβCD was negative stained and observed by electron microscope. (**E**) 293T cells were infected with sucrose-cushion-repurified virus that was treated with various concentrations of MβCD and cholesterol, and at 24 h post-infection, the cell lysate was analyzed by WB with anti-HN and anti-GAPDH Abs. (**F**) Viral titers of MβCD-treated or untreated HPIV3. Error bars, mean ± SD of three experiments. Student’s *t* test; * *p* < 0.05; ** *p* < 0.01; *** *p* < 0.001; **** *p* < 0.0001; NS, non-significant.

When considering that HPIV3 may assemble and bud in lipid rafts and cholesterol is a component of lipid rafts on the viral envelope, the relative content and the function of cholesterol on the HPIV3 envelope were detected by virions that were treated with MβCD. First, the relative cholesterol content of a virus that was treated with various concentrations of MβCD for 30 min. at 37 °C was determined by using an Amplex^TM^ Red Cholesterol Assay kit, according to the manufacturer’s protocol. The fluorescence results that were detected by the kit showed a dose-dependent drop in the level of virus cholesterol (Figure 5A). Second, the MβCD-treated virus was subjected to sucrose concentration gradient centrifugation to remove any residual drug, and the relative number of genomes of the virus were detected by RT-QPCR to rule out the possibility that the viral genome may be lost by consuming the viral envelope’s cholesterol, and our results show that the MβCD treatment produced the specific and efficient depletion of envelope cholesterol without leaking the virus genome (Figure 5B). The MβCD-treated virus was repurified through a sucrose cushion, the numbers of the HN and F proteins on the viral envelope were detected by western, and the virions were negative stained to detect the integrity of the virions to further demonstrate that the MβCD-treated virions were still intact. Our results show that the MβCD-treated virus has the same number of HN and F proteins as the untreated virions (Figure 5C). The electron micrographs also showed that MβCD-treated or untreated virions were similar in morphology, and they all had a complete membrane structure (Figure 5D). Subsequently, cells that were infected with the purified virus that were treated or mock-treated by MβCD detected the role of virus envelope cholesterol in HPIV3 infection. The western blot results show that the expression of the HN protein in cells that were infected with MβCD-treated HPIV3_F-Flag_was gradually reduced in a dose-dependent manner (Figure 5E). Viral titers were detected by counting plaque-forming units to demonstrate that the reduction in the HN protein expression is due to a reduction in viral infectivity (Figure 5F). These results suggest that the HPIV3 virus envelope contains cholesterol coming from the host cells, and the reduction of cholesterol on the viral envelope results in attenuated infectivity.

### 3.7. Cholesterol-Depleted HPIV3_F-Flag_ Particles are Defective for Internalization

**Figure 6:**
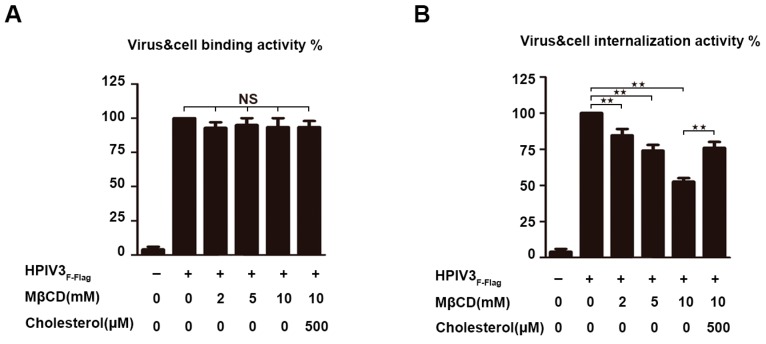
Cholesterol-depleted HPIV3 particles are defective for internalization. (**A**) Virus-cell binding assay. HPIV3_F-Flag_ virus was treated with MβCD and cholesterol as indicated and was bound to the surfaces of 293T cells on ice at a MOI of 1. Then, the cells were washed with cold PBS to remove un-adsorbed virus, and total RNA was extracted from cells by using TRIzol according to the manufacturer’s instructions. Then, RT-PCR assay was conducted to quantify the amount of cell-bound viral RNA. (**B**) Internalization assay. Cells were warmed to 37 °C and maintained for 2 h, after which they were treated with 0.25% trypsin for 10 min. at 37 °C, total RNA were extracted by TRIzol, and then RT-PCR assay was conducted to quantify the amount of internalized viral RNA. Error bars, mean ± SD of three experiments. Student’s *t* test; * *p* < 0.05; ** *p* < 0.01; *** *p* < 0.001; **** *p* < 0.0001; NS, non-significant.

It has been shown that the binding of the HN protein to the sialic acid-containing receptors initiates the infection of HPIV3, and a series of conformational changes in the F protein are induced after binding. Subsequently, fusion between the viral envelope and cell membrane occurs. Finally, the viral RNP complex is released into the cytoplasm of the target cell for a new round of virus transcription and translation. To determine whether the viral envelope cholesterol plays a role in these events, virus binding and internalization were separately measured by RT-QPCR. For this approach, the relative number of genomes of the virus that were bound to the cell surface or being internalized in the cell was used as a criterion for the ability of the virus to bind to target cells and the ability of the virus to fuse with cell membranes. The results show that the reduction of cholesterol on the viral envelope did not affect the binding of HPIV3 particles to target the cells (Figure 6A). However, a dose-dependent defection for internalization was detected, and this result indicated that cholesterol on the HPIV3 envelope plays a key role in virus internalization (Figure 6B).

## 4. Discussions

In conclusion, the results that are presented in this paper show that the F protein is able to release VLPs when it is expressed alone. The F protein has the ability to regulate the M protein’s formation of VLPs. The F protein, HN protein, M protein, and virus genome can be enriched in lipid rafts to different degrees in HPIV3-infected cells. The depletion of cholesterol on the HPIV3 envelope by incubating the virus with MβCD, which results in a significant inhibition of virus infectivity, was most likely caused by an inhibition of virus fusion.

Viral assembly and budding are critical steps in the viral life cycle. VLP systems have been proven to be practical tools in studying viral assembly and budding and determining the individual roles of different viral proteins in virion formation. For many paramyxoviruses, the M proteins drive viral assembly and budding. The M proteins of HPIV3 [24], Sendai virus (Sev) [31], Newcastle disease virus [32], and Ebola virus [33] can all be released into the culture medium when they are expressed alone; however, the Fusion protein has been reported to be an important or essential factor in virus budding, as the Nipah virus (Niv) F protein [34] and Hendra virus F protein [27] can autonomously induce the formation of VLPs. We transfected the HPIV3 F protein alone in 293T cells based on the above reports and found that the F protein can be secreted into the culture medium as VLPs. To examine which protein has a leading role in the formation of HPIV3 virions, the F protein was co-transfected with M or M_L302A_. We hypothesized that the ability of the HPIV3 F protein to regulate the VLP formation of M and M_L302A_ may be achieved by forming a complex with the M protein. Our previous study showed that the M protein of HPIV3 can recruit the N protein and P protein into VLPs [35]. We expected that both the encapsulation of the RNP complex by the M protein and the regulation of the M protein by F protein and HN protein are critical processes in the budding of HPIV3. Therefore, the regulation of the ability of the M protein to form VLPs by the F protein may be biologically significant for HPIV3 budding, possibly by regulating the rate and orientation of VLP formation of the M protein on the surface of the cell membrane.

A recombinant HPIV3_F-Flag_ with a Flag tag at the C-terminal of the F protein was constructed to study the assembly and budding of HPIV3, as we observed that the F protein of HPIV3 plays a leading role in the formation of VLPs. We found that the F protein, HN protein, and virus genome can be detected in lipid rafts in HPIV3_F-Flag_-infected cells. These results suggest that HPIV3 may use lipid rafts to recruit viral glycoproteins and virus genome for virion assembly and budding. However, we found that the use of MβCD to remove cholesterol from the cell membrane did not affect the budding and infectivity of HPIV3. There are two possible reasons for this: The first is that, although the cell membrane lipid rafts are destroyed, the residual cholesterol remains reconstituted for the virus, so the virions that are released from MβCD-treated cells still have the same infectivity as untreated cells, and the second is that HPIV3 might acquire the envelope cholesterol before it begins budding out from the cell membrane. In addition, the cytoplasm contains many cell structures accompanied by lipid rafts and enzymes that are associated with the cleavage of the F protein, and all of these provide a powerful possibility for HPIV3 to assemble and mature in the lipid rafts of the cytoplasm. If the virus assembles and germinates in the lipid rafts, it is possible for it to obtain cholesterol from the host cells, as previous studies have suggested that cholesterol on the virus envelope plays a key role in the infection of Borna disease virus [21], Human herpesvirus type 6 [19], Hepatitis C virus [22], Human immunodeficiency virus type 1 [36], Influenza virus [37], and Dengue virus [20]. The depletion of the virus envelope cholesterol leads to the inability of the viral envelope and cell membrane to fuse. As the cholesterol/phospholipid ratios of membranes affect the bilayer fluidity, the depletion of cholesterol on the virus envelope may make the envelop itself less rigid and may inhibit the conformational change in the glycoproteins that is required for fusion. Our results show that the envelope of HPIV3 contains abundant cholesterol, and cholesterol on the HPIV3 envelope was necessary for infection. The infectivity of inactivated HPIV3 by MβCD was partially rescued when the virion suspensions were treated with exogenous water-soluble cholesterol. The RT-QPCR results in Figure 6 show that the reduction of the viral envelope cholesterol mainly leads to the virus not being able to fuse with the plasma membrane without affecting the binding of the virus to the target cells. These experimental results indicate that HPIV3 is likely to assemble in lipid rafts and to acquire cholesterol on the viral envelope for effective infection.
